# Acute Effect of Dryland Maximum Strength Training Session on Sport-Specific Performance Tests in Female Water Polo Players

**DOI:** 10.3390/sports13110378

**Published:** 2025-11-03

**Authors:** Ioannis Malliaros, Gavriil G. Arsoniadis, Petros G. Botonis, Gerasimos Terzis, Theodoros Platanou, Argyris G. Toubekis

**Affiliations:** 1 Division of Aquatic Sports, School of Physical Education and Sports Science, National and Kapodistrian University of Athens, Dafne, 17237 Athens, Greece; malliasg02@gmail.com (I.M.); garsoniadis@phed.uoa.gr (G.G.A.); pboton@phed.uoa.gr (P.G.B.); tplatan@phed.uoa.gr (T.P.); 2 Sports Performance Laboratory, School of Physical Education and Sports Science, National and Kapodistrian University of Athens, Dafne, 17237 Athens, Greece; gterzis@phed.uoa.gr; 3 Division of Biology of Exercise, School of Physical Education and Sports Science, National and Kapodistrian University of Athens, Dafne, 17237 Athens, Greece

**Keywords:** water polo skills, dryland training, maximum strength training, performance

## Abstract

Background: The study evaluated the acute effect of dryland maximum strength (MS) training on water polo performance. Methods: Twelve female players (20.3 ± 1.4 years) underwent initial assessments, including a head-out 20 m swim and a one-repetition maximum (1RM) strength test in three exercises: bench press, seated pull row, and half squat. These exercises were used as the experimental (EXP) condition. During the main testing sessions, participants completed the EXP and a control (CON) condition. In the EXP, players completed MS training (three sets of six repetitions at 80% 1RM), followed 15 min later by in-water testing. In the CON, only the in-water tests were performed. These included a 10 s tethered swim to measure force, a 20 m head-out swim at maximum intensity to measure performance time, ten goal-targeted throws to reach the highest accuracy and throwing velocity, and three in-water vertical jumps as high as possible. Results: The performance time in the head-out 20 m swim (EXP: 14.21 ± 0.4, CON: 14.18 ± 0.5 s), tethered swimming force (EXP: 86.85 ± 14.82, CON: 89.58 ± 15.92 N), shooting velocity (EXP: 14.67 ± 1.19, CON: 14.91 ± 0.32 m·s^−1^), shooting accuracy (EXP: 16.5 ± 5.4, CON: 19.0 ± 5.1 points), and in-water vertical jump height (EXP: 51.7 ± 5.6, CON: 52.9 ± 4.2 cm) were no different between conditions (*p* > 0.05). Conclusions: Dryland maximum strength training performed with high loads (80% 1RM) does not impair subsequent performance during sport-specific testing in female water polo players. These findings suggest that such MS training can be safely implemented 15 min prior to in-water training sessions.

## 1. Introduction

Water polo is a physiologically demanding sport that necessitates both aerobic and anaerobic adaptations, along with the development of muscular strength and power [1]. The enhancement of physical and technical abilities in water polo, such as sprint swimming, grappling, explosive movements in and over the water, and throwing velocity, requires the development of high levels of maximum strength and muscular power [2]. Regardless of the training phase (e.g., pre-season or in-season), athletes routinely engage in dryland strength training as part of their daily regimen. These sessions typically occur in the morning or prior to water-based training and involve a range of intensities, commonly between 40% and 90% of the one-repetition maximum [1,3]. Specifically, a 16-week lower-body resistance and power-oriented training program has been shown to enhance countermovement jump capacity, maximum strength during full squat exercises, and in-water boost performance in female water polo players [3]. These adaptations have been observed during both the pre-season [1,2,3,4] and in-season periods, both in male and female water polo players [2,3,4].

Limited attention, however, has been directed toward the acute effects of maximum strength training (MS) on subsequent water polo performance tests. Specifically, Dalamitros et al. [5] found a reduced repeated-sprint performance in male water polo athletes following an MS session. In swimming, studies have demonstrated a decreased stroke length and increased stroke rate during 5 × 400 m swimming training at a pace corresponding to 4 mmol·L^−1^ blood lactate after an acute MS training compared to no strength training [6]. More recently, however, Arsoniadis et al. [7] reported comparable impairments in swimmers’ technique during a sprint training set when MS, dryland muscular endurance training, or no dryland training preceded the sprint session.

Beyond physical performance, technical skills (e.g., shooting accuracy, shooting velocity, in-water jump) are critical in water polo training and may be modified by neuromuscular or metabolic alterations, at least in male players [1]. However, female athletes may present differences in fatigability due to sex-related differences in neuromuscular activation and muscle fiber composition (i.e., a higher proportion of type I fibers) contributing to fatigue resistance [8]. This has not been examined in training for water polo. To date, although some evidence indicates reduced water polo physical performance following an MS session, it remains unknown whether, alongside physical performance, important technical abilities are also impaired.

Although water polo athletes frequently incorporate MS dryland training alongside water polo-specific sessions, limited research has examined the acute impact of such training on subsequent in-water polo-specific performance tests. This consideration may also be of substantial importance for athletes in other team or individual sports that involve similar training routines. To the best of our knowledge, there is limited evidence available to guide water polo coaches to combine dryland training sessions and specific water polo sessions in one training unit. Therefore, the present study aimed to assess the acute effects of an MS training session on water polo-specific performance and technical skills. It was hypothesized that the MS session would negatively affect 20 m sprint performance, shooting ability, and in-water vertical jump height when compared to a control condition without preceding dryland strength training.

## 2. Materials and Methods

### 2.1. Participants

Twelve female water polo players (age: 20.3 ± 1.4 years, body mass: 62.4 ± 11.1 kg, body height: 165.1 ± 7.2 cm) who compete in first division teams in the Greek Championship voluntarily participated in this study. An a priori power analysis was conducted in G*Power 3.1 (F tests; ANOVA: repeated measures, within–between interaction). Assuming a = 0.05, power = 0.88, a medium effect size (Cohen’s f: 0.40; equivalent η^2^_p_ = 0.14), correlation among repeated measures of 0.70, and non-sphericity correction ε = 1, the required total sample size n = 12 [9]. The inclusion criteria required participants to (a) demonstrate at least three years of experience in dryland resistance training, (b) be free from injury, and (c) abstain from medication intake before, during, and after the experimental procedures. The study received approval by the School of Physical Education and Sports Science review board (approval number: 1055/18/4/2018). The study was conducted in accordance with the principles outlined in the Declaration of Helsinki for research involving human subjects.

### 2.2. Experimental Approach to the Problem

A repeated-measures experimental design was used in this study. Water polo players participated in the experimental (EXP) and control (CON) conditions applied a week apart in a randomized and counterbalanced order.

### 2.3. Procedures

#### 2.3.1. Preliminary Testing and a Familiarization Session

During three consecutive sessions, 24 h apart, female water polo players participated in preliminary testing and familiarization. In the first session, players’ anthropometric characteristics, including body mass and height (Seca, Hamburg, Germany), were assessed. In the second session, participants underwent familiarization with the dryland resistance exercises that were subsequently implemented under experimental conditions. This familiarization session included the bench press, seated pull rowing, and leg press exercises, performed in 2 to 3 sets of 15 to 20 repetitions using a self-selected external load.

During the third session, the one-repetition maximum (1RM) was assessed for the bench press (ICC = 0.96), seated pull rowing (participants were allowed to move their torso during the pull; ICC = 0.98), and leg press (ICC = 0.97) exercises, following standardized testing protocols [10]. ICC values were estimated based on prior testing in a comparable population.

#### 2.3.2. Experimental Conditions

**Figure 1:**
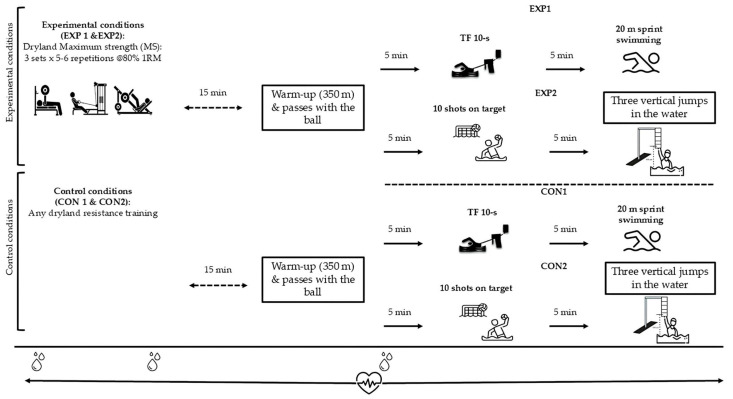
The study included four testing conditions: two experimental (EXP1 and EXP2) and two control (CON1 and CON2), each designed to evaluate performance outcomes of 20 m sprint, tethered force (TF), in-water vertical jump height, technical skills (shooting accuracy), and physiological variables (blood lactate and heart rate) under distinct training scenarios. 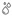: blood lactate concentration, 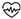: heart rate.

Following the completion of preliminary and familiarization sessions, water polo players participated in experimental conditions. To avoid accumulating fatigue when applying repeated in-water testing in the same session, we applied two experimental and two control sessions. Then, the study protocol comprised four distinct testing conditions. In the two experimental conditions (EXP1 and EXP2; see Figure 1), athletes completed an MS training session 15 min prior to undertaking the in-water performance assessments. In the two control conditions (CON1 and CON2; see Figure 1), no dryland resistance training was performed before the in-water tests. In the EXP1 and CON1 conditions, participants performed a 10 s tethered swimming sprint test followed by a 20 m front crawl sprint at maximal effort. In the EXP2 and CON2 conditions, participants completed ten target shots and three in-water vertical jumps. Recovery periods before and after testing were standardized across all conditions and lasted 5 min. Preliminary testing ensured that a 10 s tethered swimming test does not affect performance in the 20 m front crawl test.

All players were asked to provide self-reported information regarding the regularity of their menstrual cycles and the approximate onset date of menstruation in each month. This information was used to schedule all testing sessions during the mid-follicular phase of the menstrual cycle (days 7–13), as defined from the first day of menstruation. Cycle phase classification aimed to minimize the potential confounding effects of hormonal fluctuations on the measured outcomes [11].

Preliminary testing and experimental conditions were conducted in the post-season period in a 25 m indoor swimming pool with a water temperature of 26–27 °C. Water polo players performed a dryland resistance training session in an indoor gymnasium with an ambient temperature of 20–21 °C under the supervision of two certified strength and conditioning coaches. All trials were performed under both EXP and CON conditions, following a standardized in-water warm-up.

##### Dryland Resistance Training

The dryland resistance training was conducted in EXP1 and EXP2 conditions 15 min prior to the in-water warm-up and 30 min prior to the in-water performance assessments. The MS session consisted of three resistance exercises targeting both the upper and lower limbs (see Table 1). Specifically, two exercises focused on the upper body (bench press and seated pull row), while one exercise targeted the lower body (leg press). These exercises were selected based on their effectiveness and frequent application in the previous literature involving aquatic sports athletes [7]. Furthermore, the training volume of the MS session was estimated by adjusting the number of sets, repetitions, and load (%1RM) for each exercise individually (Table 1) [12]. Then, the total training volume was calculated by summing the volumes from each exercise (Table 1).

Fingertip blood samples were collected before and 2 min after the MS session and analyzed for lactate concentration (BL, Lactate Scout^+^, SensLab, GmbH, Leipzig, Germany). Heart rate (HR) was recorded before and immediately after MS session (s610i; Polar Electro Oy, Kempele, Finland) and during EXP1, EXP2, CON1, and CON2. These data were also collected at the corresponding time points during CON conditions.

##### In-Water Tests

**Figure 2:**
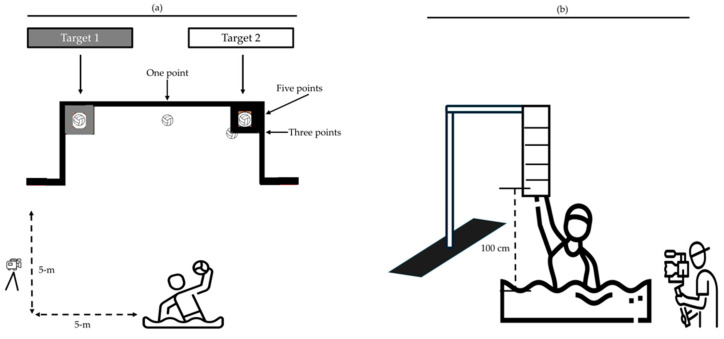
The schematic illustration of the testing procedures used to assess shooting accuracy (Panel (**a**)) and in-water vertical jump height (Panel (**b**)).

The in-water testing session was conducted 15 min after the application of MS and included a standardized 350 m warm-up in all conditions. This warm-up comprised 100 m of front crawl swimming, 100 m of individual medley swimming, 100 m of front crawl kicking, 50 m of eggbeater kicks, and 10 min of ball passing. The warm-up protocol was consistent across all experimental conditions (see Figure 2).

##### Tethered Swimming Force

The 10 s tethered swimming sprint test (TF) was used to measure specific swimming force. This procedure utilized a piezoelectric dynamometer attached to the pool’s starting block and connected to an analog-to-digital converter with a sampling frequency of 100 Hz (MuscleLab, Ergotest Innovation AS, Stathelle, Norway), thereby ensuring accurate and reliable data acquisition (ICC = 0.98) [13]. The 10 s average force value was used for the statistical analysis. Fatigue index (FI) was calculated, using force data according to Equation (1):

(1) $$FI=\left(\frac{\left(Maximum\;Force-Minimum\;Force\right)}{Maximum\;Force}\right)\times \;100$$

where maximum force and minimum force are determined as the highest and lowest force values averaged in the first and last 5 s during the 10 s tethered swimming sprint test [14]. The water polo players performed the 10 s TF test with their heads submerged, in accordance with standard swimming technique.

##### Twenty-Meter Sprint Swimming

Each participant performed a maximal-intensity 20 m front crawl swim with the head maintained above the water surface along a pre-marked 20 m lane in a standard 25 m swimming pool. The water polo players started the 20 m sprint from a position replicating the typical in-water starting movement of a water polo match, rather than by pushing off the pool wall. A high-definition digital camera (Samsung Full HD, 1920 × 1080 pixels, 20 Hz; Samsung Electronics CO., Ltd.; Suzhou; China) was positioned parallel to the swimming lane and operated by an experienced investigator walking alongside the swimmer to ensure consistent framing and capture of the entire sprint. The starting and finishing points were defined by the exact moment the swimmer’s head crossed the 20 m mark. These events were identified during post-processing using frame-by-frame analysis in a free video-editing software (Kinovea 0.7.10), allowing precise calculation of elapsed time to the nearest 0.05 s. Before the commencement of the main experimental procedures, a pilot study was conducted to determine the test–retest reliability of the 20 m maximal head-up swimming protocol, yielding an ICC of 0.82.

##### Shooting Accuracy

Female water polo players were instructed to execute ten shots aimed at designated targets (see Figure 2a). Players were located 5 m away from the target and they self-selected their target. Shooting accuracy was assessed based on the ball’s contact with the target. Specifically, shots that passed cleanly through the target without any contact were awarded five points; those involving minimal contact with the target were awarded three points; and shots that failed to pass through the target received one point [15]. The shot velocity was quantified using high-speed video analysis: a high-definition digital camera (Samsung Full HD, 1920 × 1080 pixels, 20 Hz; China) was securely mounted at a fixed position, five meters from the shooter’s location, and aligned perpendicular to the intended ball trajectory. From recorded footage, frame-by-frame analysis identified the precise moment of ball release—defined as the initial departure of the ball from the hand—and the instant of impact with the target using a free video-editing software (Kinovea 0.7.10). A 2 m calibration line, visible during recordings, was used to calculate the exact shooting distance. The mean shooting velocity derived from the ten shots was calculated and included in the statistical analysis.

##### In-Water Vertical Jumps

Three in-water vertical jumps were performed using a specially designed and calibrated measurement scale (see Figure 2b). For the evaluation of each jump, the athlete positioned themselves beneath the scale and executed the jump while being recorded from a posterior angle. The final jump height was determined by calculating the total distance from the water surface to the highest point reached by the athlete’s finger, minus the length of the athlete’s upper limb [16].

### 2.4. Statistical Analyses

Normal distribution of the data was examined using the Kolmogorov–Smirnov test. Sphericity was verified using Mauchly’s test. When the assumption of sphericity was violated, the significance of F ratios was adjusted according to the Greenhouse–Geisser correction. A two-way repeated-measures analysis of variance (conditions x time points) was then performed to compare the outcomes of the three in-water vertical jumps and the ten shots on targets between EXP2 vs. CON2. A two-way analysis of variance was employed to examine differences in HR and BL. A Tukey honest significance difference post hoc test was used to compare means when significant F-ratios were found. Moreover, a paired sample T-test was used to compare the means for time in 20 m, tethered swimming force, and fatigue index between EXP1 vs. CON1. The statistical package SPSS (v. 23) was used for the statistical analysis.

The partial eta squared (η^2^_p_) was used to calculate the effect size, which was considered small (≤0.06), medium (≤0.14), and large (≥0.14) [17]. Intraclass correlation coefficient using a one-way random effect was used to test the reliability of 1RM in bench press, seated pull row, and leg press exercises, as well as in the 20 m sprint test. Data are presented as mean and SD. Statistical significance was set at *p* < 0.05.

## 3. Results

### 3.1. Swimming Performance and TF

**Figure 3:**
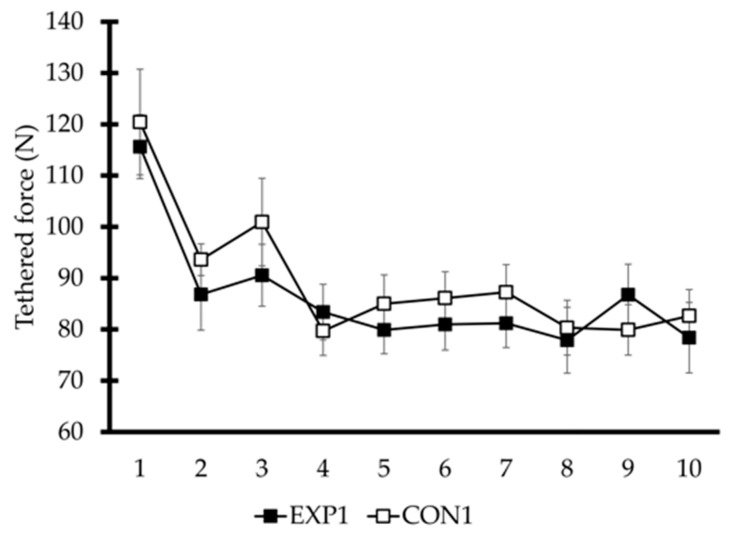
Tethered swimming force (TF) was assessed during the 10 s tethered swimming test under experimental (EXP1) and control conditions (CON1).

Swimming performance during the 20 m sprint did not differ between conditions (EXP1: 14.21 ± 0.50 s; CON1: 14.18 ± 0.40 s; *t* = 0.28, *p* = 0.78). Similarly, the 10 s TF values were comparable between EXP1 and CON1 (F_1,10_ = 1.11, η^2^ₚ = 0.10, *p* = 0.30; see Figure 3). Furthermore, FI was similar between conditions (EXP1: 33.1 ± 13.0%; CON1: 32.8 ± 12.1%; *t* = 0.30, *p* = 0.80; Figure 3).

### 3.2. Shooting Accuracy and In-Water Vertical Jumps

**Figure 4:**
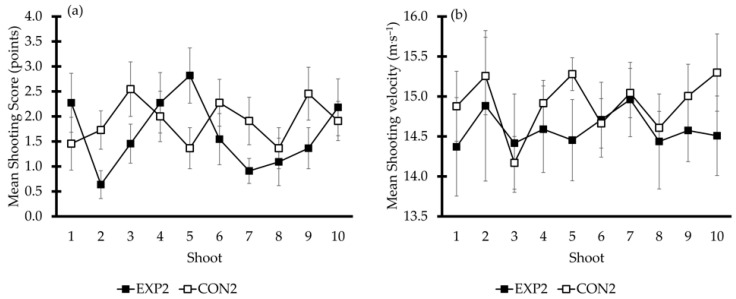
Panel (**a**) presents the mean accuracy score ± SD achieved in each one of the 10 shots, while panel (**b**) illustrates the shooting velocity recorded in each one of the 10 shooting attempts under experimental condition 2 (EXP2) and control condition 2 (CON2).

The summary of shooting accuracy after the 10 shots did not differ between conditions (EXP2: 16.54 ± 5.4 points; CON2: 19.0 ± 5.1 points; *t* = −1.31, *p* = 0.21) or within repeated shots (F_1,10_ = 1.74, η^2^ₚ = 0.14, *p* = 0.22; see Figure 4a). Similarly, no significant difference was observed in mean shooting velocity between conditions (EXP2: 14.67 ± 1.19 m·s^−1^; CON2: 14.91 ± 0.32 m·s^−1^; *t* = −0.59, *p* = 0.57) or within repeated shots (F_1,6_ = 0.62, η^2^ₚ = 0.09, *p* = 0.46; see Figure 4b).

The mean in-water vertical jump was similar between conditions (EXP2: 51.7 ± 5.6 cm; CON2: 52.9 ± 4.2 cm; *t* = −1.12, *p* = 0.29) and repeated jumps (F_1,10_ = 1.25, η^2^ₚ = 0.11, *p* = 0.29).

### 3.3. Blood Lactate Concentration and Heart Rate

**Figure 5:**
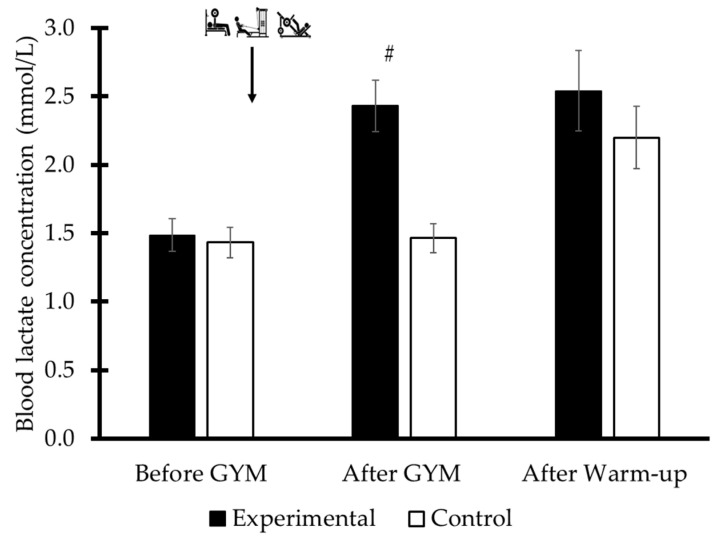
The mean blood lactate concentration was measured during the experimental and control conditions at three time points: prior to the dryland maximum strength training (Before GYM), immediately after the strength training session (After GYM), and following the in-water warm-up (After Warm-up). #: *p* < 0.05, between conditions.

The blood lactate concentration of the combined EXP1, EXP2 and CON1, CON2 was higher in EXP compared to the CON conditions (F_1,10_ = 9.78, η^2^ₚ = 0.49, *p* = 0.01) and an interaction of conditions by repeated sampling was evident (F_2,20_ = 7.07, η^2^ₚ = 0.41, *p* = 0.01). BL was higher in EXP compared to CON after the completion of the dryland session (F_2,20_ = 7.06, η^2^ₚ = 0.41, *p* = 0.01; Figure 5).

The combined heart rate was higher in EXP compared to CON only after the shooting accuracy test (EXP: 149 ± 3 b·min^−1^ vs. CON: 145 ± 2 b·min^−1^, η^2^ₚ = 0.86, *p* = 0.01).

## 4. Discussion

The aim of this study was to examine the effects of a dryland MS training session on water polo-specific performance, technical skills, and physiological responses. Technical skills were assessed through measures of shooting accuracy, shooting velocity, and in-water vertical jump performance, while muscular function was evaluated via a 10 s TF sprint. The findings revealed no significant differences in performance outcomes, tethered swimming force, shooting accuracy, shooting velocity, or in-water vertical jump height between the experimental conditions (EXP1, EXP2) and the control conditions (CON1, CON2). However, BL concentration and HR responses were higher in the experimental compared to control conditions only after the MS training.

In the present study, a 20 m distance was selected based on evidence indicating that elite female water polo players frequently cover such short distances during the game [18]. The MS session implemented in the present study yielded a comparable 20 m sprint performance time between the experimental and control conditions. These findings are consistent with previous studies reporting similar sprint outcomes following dryland strength training sessions for distances of 20 m and 40 m [4,19]. However, those studies involved either mixed-gender samples or exclusively male participants. When sprint sets (e.g., 4 × 50 m or 8 × 25 m) were performed after an MS session, similar to those applied in the present study, a decline in performance was observed in repeated sprints overall, but this was not evident in the first sprints [5,7]. This observation is consistent with the present findings. It is possible that the duration of the 20 m sprint (approximately 13–15 s) was insufficient to induce detectable changes in performance. Additionally, the specific characteristics of the MS session—such as the relatively low training volume (i.e., three exercises) and extended rest intervals (5 min)—may not be adequate to induce fatigue. This has been demonstrated in a recent study, which highlighted that strength training may exert only a limited impact on performance measures, such as the countermovement jump [20]. Additionally, athletes may be able to tolerate a higher internal load without experiencing performance impairments following an MS session [21].

Although female water polo players exhibited elevated blood lactate concentrations following the MS session, neither sprint performance nor in-water TF were adversely affected. These findings agree with previous studies reporting similar TF values during a 10 s TF sprint following MS or control sessions [6,7]. The players’ high training status and the moderate post-exercise lactate concentrations (2–4 mmol/L) may have allowed a fast recovery rate, despite the large effect sizes shown in blood lactate concentration. This has also been demonstrated in a previous study, where an increase in blood lactate concentration was observed immediately following an MS session; however, within 40 min, lactate levels had returned to resting values [22]. However, no differences were observed in the single 10 s tethered sprint, the unchanged FI, and a 20 m effort, suggesting that muscular function remained unaffected [23,24,25,26]. The application of a repeated sprint test might have revealed potential metabolic effects of the preceding MS session, and this is likely a limitation of our study.

In the present study, water polo athletes performed the swimming tests approximately 30 min after completing the MS session, and an in-water warm-up was included. However, the blood lactate concentration was similar at the start of testing in both EXP and CON conditions. Although the low lactate values of ~2.5 to 3.0 mmol/L are not adequate to impair muscular function, such increments were evident with different exercise modes. In the EXP conditions, blood lactate was increased by the MS session, while in the CON condition the increment was evident by the in-water warm-up. However, these similar metabolic effects may not have the same effect on neuromuscular function and should not be considered equal. A likely replacement of one over the other cannot be suggested with the present findings.

Although we expected impaired technical performance following the MS session, no differences were observed in shooting accuracy, shooting velocity, or in-water vertical jump height between EXP and CON conditions, aligning with previous findings [27,28]. Maximum strength training is known to induce both central and peripheral fatigue [29,30], with effects potentially persisting for 20 to 40 min post-exercise [22]. This was not evident in the present study and may be attributed to the low volume of MS sessions and long recovery periods applied within the session. Moreover, the performance assessments were conducted approximately 30 min after the MS session. Thus, the MS content and the long recovery period, combined with the nature of the in-water tests, minimized any potential interference effect.

Recent evidence suggests that, when maximum strength training sessions are followed by high-intensity low-duration efforts (ranging from 3 to 30 s), performance is generally maintained without observable decrements [31]. Nonetheless, it is important to acknowledge that the duration and structure of the performance tests employed in the current study, all with short duration, may evaluate neuromuscular potential but do not maximally stress the metabolism. Thus, it does not allow for the detection of more subtle impairments, and this may be a potential limitation of the study. The tests applied in the present study may stress the alactic anaerobic metabolism (phosphocreatine: Pcr). However, this metabolic pathway recovers fast, and this is independent of the previous neuromuscular stress [20,30,32].

It should be acknowledged that the relatively small sample size may limit the generalizability of the findings. Second, the participants were exclusively elite first-division female water polo players competing in the Greek Championship. While this provides valuable insight into a high-performance population, the results may not be directly applicable to athletes from lower competitive divisions, younger age categories, or male counterparts.

## 5. Conclusions

A dryland resistance training session including three upper- and lower-body exercises executed in 3 sets of 5–6 repetitions at 80% of the one-repetition maximum, with a 5 min rest interval between sets, can be implemented in female elite water polo athletes prior to in-water training without impairing shooting accuracy, velocity, or in-water vertical jump ability. Additionally, in-water force, as well as blood lactate concentration and heart rate responses, were similar between EXP and CON conditions. To the best of our knowledge, this is the first study to investigate these effects specifically in female water polo athletes. Further research is warranted to explore the acute influence of dryland resistance training incorporating varying characteristics—such as exercise selection, intensity, rest intervals, training volume, and targeted muscle groups—as well as different recovery durations prior to in-water sessions.

## 6. Practical Application

Based on the present findings, it is recommended that water polo coaches may consider incorporating dryland maximum strength training prior to in-water performance tests of short duration. Furthermore, this type of MS training may be strategically applied before water polo training sessions that emphasize the development of players’ technical skills, as it appears not to impair subsequent performance. Such an approach may allow coaches to integrate strength development within regular training routines without compromising sport-specific outcomes.

## Figures and Tables

**Table 1:** Detailed description of the dryland maximum strength training session applied 15 min before the in-water tests.

Exercises	Number of Sets	Number of Repetitions	Load (%1RM)	Rest	Training Volume (kg)
Bench press	3	5–6	80	5 min	1440
Seated pull row	3	5–6	80	5 min	1440
Leg press	3	5–6	80	5 min	1440
Overall duration	45 min				
Overall training volume	4320 kg				

## Data Availability

Data could be available upon reasonable request to the corresponding author.
